# Review: Research progress of adipose-derived stem cells in the treatment of chronic wounds

**DOI:** 10.3389/fchem.2023.1094693

**Published:** 2023-02-13

**Authors:** Zhuolun Hao, Wenli Qi, Jiaming Sun, Muran Zhou, Nengqiang Guo

**Affiliations:** Department of Plastic Surgery, Union Hospital, Tongji Medical College, Huazhong University of Science and Technology, Wuhan, China

**Keywords:** adipose-derived stem cells, chronic wounds, healing, immunoregulationd, mechanism

## Abstract

Although methods are used to treat wounds clinically, there are still many challenges in the treatment of chronic wounds due to excessive inflammatory response, difficulties in epithelialization, vascularization, and other factors. With the increasing research on adipose-derived stem cells (ADSCs) in recent years, accumulating evidence has shown that ADSCs scan promotes the healing of chronic wounds by regulating macrophage function and cellular immunity and promoting angiogenesis and epithelialization. The present study reviewed the difficulties in the treatment of chronic wounds, as well as the advantages and the mechanism of ADSCs in promoting the healing of chronic wounds, to provide a reference for the stem cell therapy of chronic wounds.

## 1 Introduction

With an increasingly aging population and soaring chronic metabolic diseases such as diabetes increase, chronic injuries have become an increasingly serious public health concern. Chronic wounds refer to wounds that cannot pass through a normal, orderly and timely repair process or the repair process fails to restore normal anatomical structure and function after 2 months (Salvo and Sandoval, 2022). Conventional wound repair methods include skin transplantation, transfer of skin shreds, application of wound dressings, and tissue engineering skin. However, these methods present problems such as transplantation necrosis, scarring, and pigmentation abnormalities (Buote, 2022). In recent years, the application of certain biologically active substances, such as growth factors and platelet-rich plasma, has reinforced the effect of traditional treatment methods. However, these substances have a short duration of action and often need to be used multiple times (Erratico et al., 2022), which increases the cost of treatment and reduces patient compliance. At present, adipose-derived stem cells (ADSCs) have been confirmed to have a significant effect in promoting the healing of chronic wounds, and have several advantages, showing a good application prospect in the treatment of chronic wounds (Liu et al., 2021).

## 2 Common risk factors of chronic wounds and challenges in their treatment

### 2.1 Common risk factors of chronic wounds

Clinically, there are several risk factors for chronic injury. The main risk factors are discussed below.

Older patients are more susceptible to chronic wounds. Because aging is associated with a decrease in cellular activity, elderly patients are less responsive to platelet derived growth factor (PDGF), which promotes the growth of wound blood vessels (Cerny et al., 2021). In addition, wound healing deteriorates with altered expression of transforming growth factor (TFG) receptors and overexpression of matrix metalloproteinases in aging cells (Mast and Schultz, 1996).Besides, unlike young skin cells, older skin cells cannot produce enough extracellular matrix (ECM), older skin cells cannot (Lobmann et al., 2005). The above mechanisms may be the reasons for the poor healing of wounds among the elderly.

Wounds in patients with underlying diseases are more difficult to heal. For example, in diabetic patients, high blood sugar reduces the solubility of the ECM, which contains multiple cytokines that promote wound healing, which indirectly leading to poor wound healing (Pradhan et al., 2009). In addition, there is a persistent inflammatory response in the wounds of diabetic patients, which is reflected in the majority of neutrophils and macrophages in wounds, complicating the normal healing process (Lobmann et al., 2005).

Nutritional status is one of the key factors affecting wound healing. Due to surgery and trauma, patients with wounds continue to be in a state of high metabolism, which leads to excessive consumption of fat and protein stored in the body, resulting in malnutrition (Grada and Phillips, 2022). The lack of proteins directly leads to the deposition of collagen on the wound surface (Fullana et al., 1993). Specifically, the lack of arginine and methionine is directly related to the difficulty associated with poor wound healing, and the main mechanisms involved are the prolongation of the inflammatory phase and the blockage of angiogenesis (Haydock and Hill, 1986).

### 2.2 Challenges in the treatment of chronic wounds

Currently, there are numerous challenges in the treatment of chronic wounds, mainly due to the uncontrollable inflammatory response at the wound site, the blocked vascularization process, the difficulty in normal epithelialization and dermal remodeling, and the adverse effects of wound microbes and aging organisms. The underlying diseases that are most likely to cause chronic wounds, such as diabetic foot ulcers, venous leg ulcers, and pressure ulcers, can lead to the above pathological changes and cause chronic wounds (Heydari et al., 2022).

The uncontrollable inflammatory reaction is one of the main challenges in the treatment of chronic injuries. Studies have shown that various of inflammatory cells, such as neutrophils and macrophages, persist on the wound site. An increase in the infiltration of these inflammatory cells may suppress the anti-inflammatory functions of normal immune cells, such as M2 macrophages (Herrick et al., 1992).

Vascularization is the only normal route of wound repair, and inadequate vascularization also complicates the treatment of chronic wounds. Chronic wounds are difficult to vascularize due to the imbalance between the promotion and inhibition of angiogenesis. Furthermore, it is difficult for macrophages to change from M1-type macrophages that stimulate inflammation to M2-type macrophages that promote repair, resulting in insufficient secretion of human vascular endothelial growth factor (hVEGF) and other pro-angiogenic factors by M2-type macrophages(Michaels et al., 2007). The promotion of normal vascularization of wounds remains a major challenge in the treatment of chronic wounds.

The complexity of normal re-epithelialization and dermal remodeling of chronic wounds is another challenge in the treatment of wounds. A study found that the expression of β-catenin was increased at the edge of chronic wounds of a diabetic foot. *In vitro* studies have shown that β-catenin blocks the action of epidermal growth factor, thereby inhibiting wound healing (Pastar et al., 2010). Microorganisms in chronic wounds also have a direct impact on wound repair. In the treatment of chronic wounds, biofilms formed by various microorganisms lead to resistance of wound microorganisms to traditional antibiotics and host defenses (Diridollou et al., 2001).

## 3 Potential mechanisms of ADSCs in the treatment of chronic wounds

Wound healing is a continuous and complex process that is critical to maintaining the barrier function of the skin. Skin wound healing is a complex and orderly dynamic physiological response process produced by the body after injury, which often includes three stages: inflammation, cell proliferation and migration, and tissue remodelling. In recent years, studies have found that ADSCs play a therapeutic role by acting on multiple aspects of wound healing.

**FIGURE 1:**
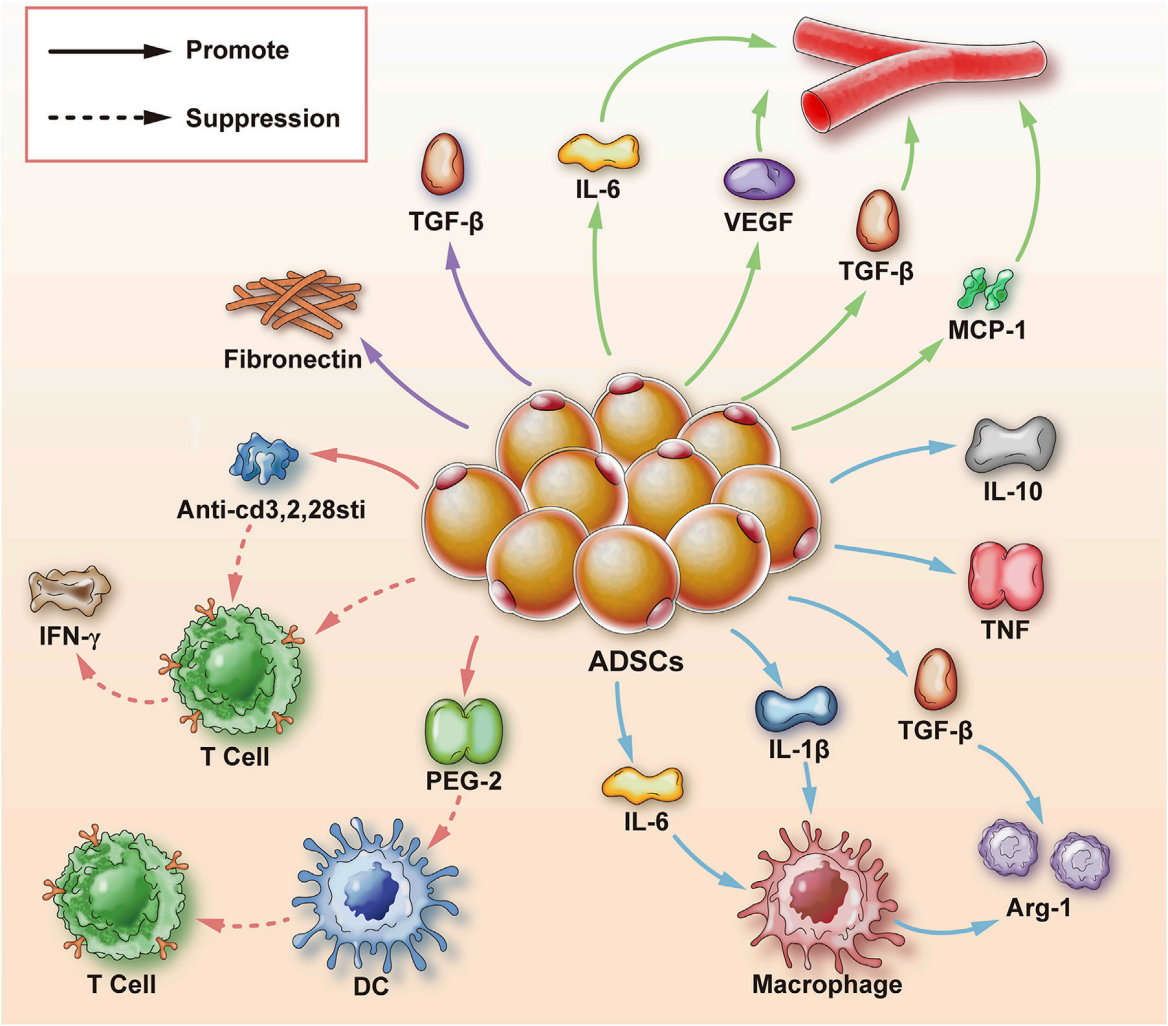
Mechanism of adipose-derived stem cells to promote wound healing. ADSCs are adipose-derived stem cells; Arg-1 is arginase-1; TGF-β is transforming growth factor-β; IL-1β, 6, 10 is interleukin-1β, 6, 10; TNF is tumor necrosis factor; MCP-1 is monocyte chemoattractant protein-1; hVEGF is human vascular endothelial growth factor; T cell is T cell; DC is dendritic cell; PGE-2 is prostaglandin-2; IFN-γ is Interferon γ; Anti-cd3, 2, 28sti are anti-CD3, CD2, CD28 stimulators; fibronectin generally refers to fibronectin; solid arrows represent promotion; dashed arrows represent inhibition.

The mechanisms by which ADSCs promote wound healing can be roughly divided into two aspects. The first is the differentiation function of ADSCs which can differentiate into fibroblasts, vascular endothelial cells, nerve cells, keratinocytes, skeletal muscle cells, chondrocytes, and cardiomyocytes. The second is the exocrine function of ADSCs. ADSCs can secrete pro-angiogenic cytokines such as hVEGF, thereby regulating angiogenesis and other activities to promote wound healing. ADSCs can also secrete a variety of cytokines to regulate macrophages and cellular immunity as well as exosomes to regulate the wound-healing process (Xiong et al., 2020) (Figure 1).

### 3.1 Regulation of macrophages by ADSCs

One of the reasons for the formation of chronic wounds is that the wounds remain in the inflammatory response stage, and macrophages can play an important role in the inflammatory response. ADSCs can regulate the aggregation, number, and type of macrophages by secreting various cytokines, ultimately regulating macrophages to promote wound healing. M1 is the predominant macrophage type in the tissue during wound repair and inflammation, which removes pathogens such as bacteria and viruses. With the progress of wound repair, M1 macrophages begin to transform into anti-inflammatory macrophages (M2-type) (Fantin et al., 2010). However, it is difficult for macrophages to change from M1 to M2 when the body has factors such as low immunity, hormone level disorder, high glucose environment, etc., and the balance between pro-inflammatory and anti-inflammatory is broken, resulting in a long-term inflammatory state of the wound and the formation of chronic wounds (Borthwick et al., 2016). Specifically, in high glucose environment, there are 13 pro-inflammatory factors upregulated including tumor necrosis factor α(TNFα), interleukin −1 (IL-1), IL-6. Because M1 macrophages are more active in a hyperglycemic environment, and they more difficult to polarize into M2 macrophages (Morey et al., 2019). Besides, previous experiments have shown that the conditioned medium of ADSCs can stimulate macrophages and increase the secretion of anti-inflammatory cytokines such as TNF and IL-10, thereby stimulating wound healing (Stojanović and Najman, 2019). The study found that when ADSCs were co-cultured with macrophages, ADSC exosomes induced an increase in the number of M2 macrophages, leading to higher levels of type I arginase Argininemia-1, (Arg-1) (Sin et al., 2017). The inflammatory response of cells regulates the inflammatory reaction and metabolic homeostasis and facilitates wound healing (Zhao et al., 2018).

### 3.2 Regulation of cellular immunity by ADSCs

Refractory wounds pose a significant challenge for clinical intervention and can be formed for many reasons. For instance, the immune response is gradually weakened without negative feedback after the inflammatory period, resulting in the persistence of immune cells, and the abnormal existence of immune cells impairs tissue healing, which seriously affects the speed of tissue repair (Gieseck et al., 2018). ADSCs can reduce the inflammatory response through direct cell contact and secretion of cytokines, and play a role in regulating immune strength to promote wound healing. The effect of T lymphocytes on cellular immunity has been confirmed. Studies have shown that prostaglandin E2 (PGE2) from ADSCs is a regulatory factor that inhibits anti-inflammatory presenting cells; dendritic cells overmature to attenuate T lymphocyte proliferation and cytokine secretion, thereby attenuating the inflammatory response (Yañez et al., 2010). In addition, studies have shown that ADSCs have an inhibitory effect on T cell differentiation and activation, and reduce *in vitro* expansion of T cells by phenotypic characterization and functional analysis of cytotoxic and helper T cell (activation and differentiation marker T cells) proliferation and interferon release (Blazquez et al., 2014). Other studies have shown that ADSC exosomes can also inhibit the differentiation of CD4^+^ and CD8^+^ T cells into their effector or memory cell phenotypes by mediating anti-CD3, CD2, and CD28 stimuli, reduce inflammation, and promote wound healing (Ren et al., 2019).

### 3.3 ADSCs promote angiogenesis

Mounting evidence suggests that ADSCs are involved in neovascularization in tissue repair and wound healing. Local angiogenesis affects two aspects. First, the rich blood supply can provide nutrients and oxygen to the wound. Second, the rich vascular network plays an important role in absorbing necrotic substances and controlling local wound infection (Guan et al., 2021). Studies have shown that the promotion of angiogenesis is mainly achieved by secreting and regulating the activities of various cytokines. hVEGF and TGF-β secreted by ADSCs are closely associated with angiogenesis (Hsu et al., 2019). Besides hVEGF, the cytokine IL-6 secreted by ADSCs also has a positive effect on angiogenesis. In a study comparing IL-6 knockout mice and normal mice, it was found that wound healing was delayed in IL-6 knockout mice. It is speculated that IL-6 may play a direct and critical role in wound healing by promoting angiogenesis (Lin et al., 2003). Even in severe limb ischemia, ADSCs can still prevent vascular endothelial cell apoptosis and induce neovascularization through secreted growth factors and anti-inflammatory cytokines, indicating that ADSCs have a wide range of effects in promoting wound vascularization (Liang et al., 2018).In addition to the indirect effects of cytokines, ADSCs can also interact directly with vascular endothelial cells and macrophages, increasing the secretion of human macrophage chemoattractant protein-1 (MCP-1) and hVEGF, thereby promoting angiogenesis (Bachmann et al., 2020).

### 3.4 ADSCs promote epithelialization

Epithelialization is an important marker of wound healing, and only complete epithelialization of the wound can prevent water loss and resist pathogenic infection. According to previous studies, chronic epithelialization of wounds was complicated by bacterial infections and inflammatory responses (Li et al., 2018). In terms of bacterial infections, the bacteria that cause delayed wound healing are mainly Gram-positive bacteria, such as *Staphylococcus aureus* and *Streptococcus* pyogenes. As far as the severity of bacterial infection is concerned, it is related to the following aspects: the duration of infection, the virulence of bacteria, the site of infection and the strength of the body’s immunity(Hawn et al., 2011). After treatment with ADSCs, the expression of fibronectin in the wound tissue was increased, and fibronectin ultimately promoted the epithelialization of the wound by recognizing the arginine-glycine-aspartic acid sequence of the α5β1 protein receptor of skin epithelial cells (Zang et al., 2019). In addition, studies have shown that adipose tissue extracellular fractions containing ADSCs isolated from lipoaspirate also have the potential to promote wound epithelization, in which adipose tissue extracellular fractions enhanced dermal and epidermal cell proliferation in a dose-dependent manner, thereby promoting epithelization (Bellei et al., 2018).

### 3.5 ADSCs regulate ECM synthesis

The ECM plays a skeletal role in wound healing, thereby accelerating the formation of scars and shortening the time of wound healing. In wound repair, an ECM consisting of type I collagen forms a scar that temporarily seals the wound (Verhaegen et al., 2009). However, in the subsequent process, the ECM is gradually degraded by matrix metalloproteinases, which makes the wound difficult to heal (Keskin et al., 2021). ADSCs have been shown to regulate the ECM remodeling process. There is an orderly increase in the deposition of type III collagen deposition at the site of the wound site and regeneration of the skin appendages in wounds treated with ADSCs. Specifically, ADSCs accelerate the remodeling of the ECM during wound repair by up-regulating the ratio of type III collagen to type I collagen, thereby promoting wound healing (Wang et al., 2019) (Table 1).

**TABLE 1:** Summary of the mechanism of ADSCs promoting wound healing. ADSCs are adipose-derived stem cells; TGF is transforming growth factor; IL is interleukin; TNF is tumor necrosis factor; Arg is arginase; PGE is prostaglandin; DC is dendritic cell; IFN is tumor necrosis factor; VEGF is Vascular endothelial growth factor; MCP is monocyte chemotactic protein; AT-Ex adipose tissue extracellular fraction.

Different mechanisms of ADSCs promoting wound healing	Method	References
regulation of macrophages	Secretes TGF-β, IL-1β and IL-6 and increases macrophage recruitment	Deng et al. (2019)
	Secretion of TNF, IL-10	Stojanović and Najman (2019)
	Induced synthesis of Arg-1	Sin et al. (2017)
regulation of cellular immunity	Secretion of PGE-2 inhibits DC hypermaturity and attenuates T cell proliferation	Yañez et al. (2010)
	Inhibit T cell differentiation and activation, reduce IFN-γ	Blazquez et al. (2014)
	Mediates anti-CD3, CD2, CD28 stimuli, inhibits T cell activation	Ren et al. (2019)
angiogenesis	Secretion of VEGF, TGF-β, IL-6	Lin et al. (2003)
	Interacts with vascular endothelial cells and macrophages to increase MCP-1 and VEGF secretion	Bachmann et al. (2020)
epithelialization Regulate the extracellular matrix	Recognizing the RGD sequence of the α5β1 protein receptor	Zang et al. (2019)
	Enhances dermal and epidermal cell proliferation in a dose-dependent manner Upregulates the ratio of collagen type III to collagen type I	Bellei et al. (2018)
		Wang et al. (2019)

## 4 Strengths and weaknesses of ADSCs

**FIGURE 2:**
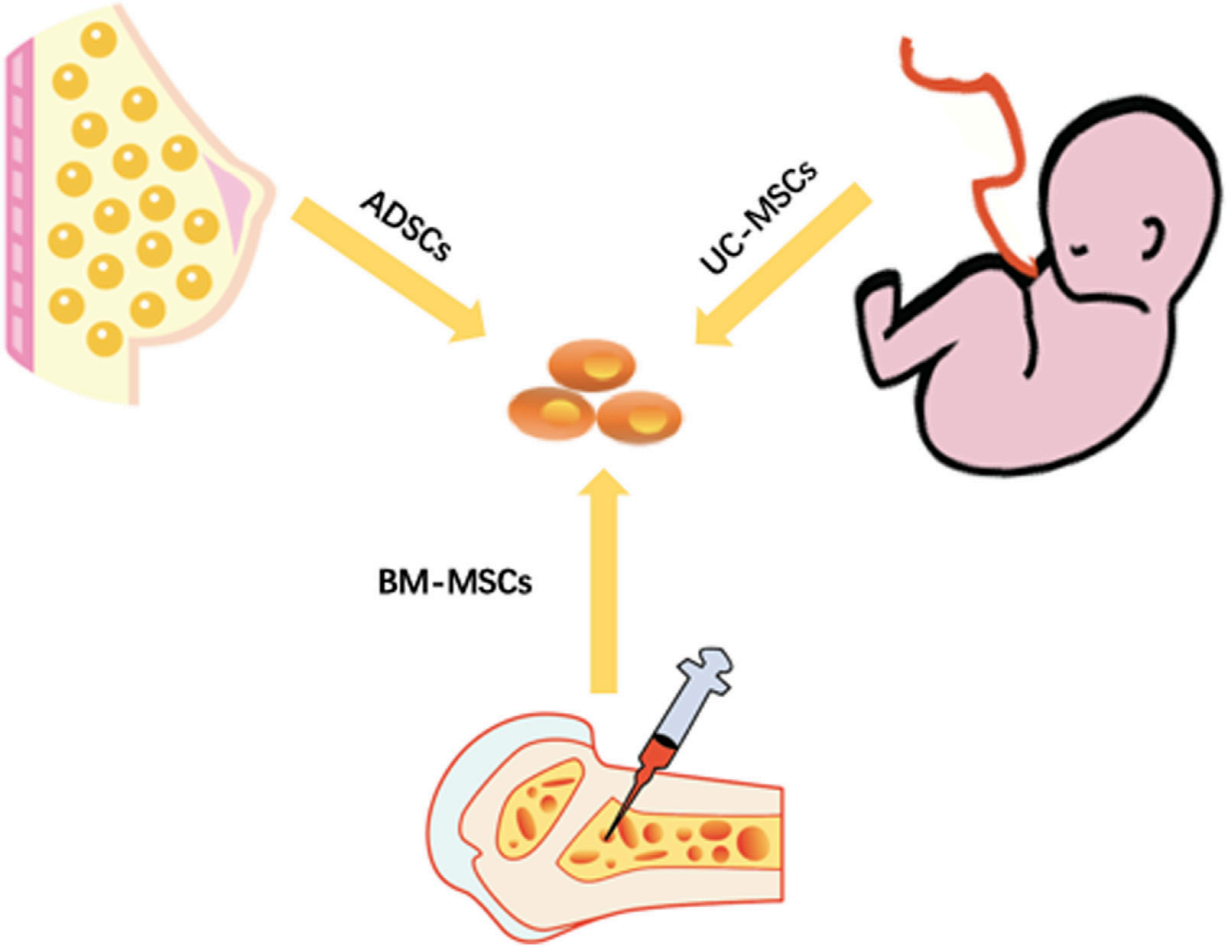
Three main sources of stem cells. ADSCs are adipose-derived stem cells. UC-MSCs are umbilical cord mesenchymal stem cells. BM-MSCs are bone marrow mesenchymal stem cells.

Mesenchymal stem cells(MSCs) can be derived from various of tissues such as bone marrow, fat, placenta, umbilical cord, umbilical cord blood, amniotic membrane, and dental pulp (Liu et al., 2022). Therefore, MSCs can be divided roughly into ADSCs, bone marrow-derived mesenchymal stem cells(BM-MSCs), and umbilical cord-derived mesenchymal stem cells(UC-MSCs) (Figure 2).

For the acquisition site, BM-MSCs can only be obtained from the bone marrow, and the success of the acquisition and the number of acquisitions depend on the patient’s condition and the amount of bone marrow aspiration (Caplan, 2007). UC-MSCs can only be extracted from the umbilical cord of newborns, and the site is relatively single and limited (Balzano et al., 2019). ADSCs can be extracted from fat-rich sites such as the abdomen and breasts. Compared with other sources of MSCs, ADSCs can be obtained from more abundant sites (Khazaei et al., 2022). From a functional point of view, BM-MSCs are greatly affected by the patient’s age. The number of extraction and the differentiation potential tend to decline with an increase in age (Azizi et al., 2022). However, UC-MSCs are suspected to have gender differences, which limits their application range, and their differentiation ability remains controversial (Abbaspanah et al., 2021). ADSCs can maintain high cell viability and low immunogenicity for a long time. In addition, the viability of extracted ADSCs dose not decrease with age. ADSCs are transplantable in both autologous and allogeneic settings (Mazini et al., 2020).

There are some reports on the application of ADSCs in the treatment of chronic wounds. Deng et al. conducted a randomized controlled trial to compare ADSC injection wounds, comparing ADSCs injection combined with standard dressing therapy and standard dressing therapy alone (Deng et al., 2018). The results showed that the average weekly wound healing rates of the experimental group and the control group were 34.55% and 10.16%, respectively. Moreover, Tanios et al. showed that 92% of the chronic wounds in the experimental group (ADSC treatment) were completely healed, compared with only 60% in the control group (standard dressing treatment), and the healing time of the experimental group (7.87 weeks) was shorter than that of the control group (13.87 weeks).

However, the application of ADSCs in wound healing has several limitations. Currently, the application of allogeneic ADSCs involves cryopreservation of ADSCs. However, guidelines for optimal and standardized parameters for cryopreservation of ADSCs are largely lacking, and therefore contamination-free and a sufficient number of usable ADSCs cannot be guaranteed (Qayyum et al., 2017). In addition, it has been shown that ADSCs enhance breast tumor cell migration (Challapalli et al., 2021).

## 5 Conclusion and outlook

The healing process of chronic wounds is very complex, and ADSC therapy shows great advantages and potential in the promotion of healing. ADSCs can directly differentiate into fibroblasts, keratinocytes, and other cell types to promote the recovery of the shape and function of the wound by accelerating the wound repair process and regulating the wound and its surrounding microenvironment. In addition, recent studies have found that ADSCs have multiple signal recognition molecules on the cell membrane, which can be used as potential carriers for drug delivery and can be further modified by ADSC applications such as co-incubation, freeze-thaw, and electroporation to carry therapeutic molecules. Therefore, ADSC therapy is an effective and promising method to promote wound healing. Although the experimental application of ADSCs in patients with chronic wounds patients has yielded preliminary results, there are still several problems to be solved before large-scale clinical application on a large-scale. From an overall perspective, there are only small-scale experimental applications of ADSCs in the treatment of chronic wounds currently, making it difficult to obtain higher-level evidence supporting for the safety and efficacy of ADSCs. From the perspective of ADSCs collection and processing, whether the age, gender, body mass index, or fat collection site of enrolled subjects has an impact on the number and viability of ADSCs also warrants further exploration. In addition, the potential tumorigenicity of ADSCs also requires conattention. Therefore, larger-scale randomized controlled clinical trials with unified reporting standards and more in-depth basic research are required to assess safety and efficacy of ADSCs in promoting chronic wound healing.
